# Supplementary material for: High Rates of Liver Cirrhosis and Hepatocellular Carcinoma in Chronic Hepatitis B Patients with Metabolic and Cardiovascular Comorbidities
Source: Microorganisms. 2021 Apr 30;9(5):968. doi: 10.3390/microorganisms9050968 (PMC8146494; doi:10.3390/microorganisms9050968)
Supplement: Supplementary file 1 [file microorganisms-09-00968-s001.zip › microorganisms-1195055-supplementary.pdf]

Supplementary Table S1. Correlation between baseline parameters and occurrence of total events or HCC in cirrhosis patients (n=50) during follow-up analyzed by univariate und multivariate logistic regression. HCC= hepatocellular carcinoma, CHD= coronary heart disease, GGT= gamma-glutamyltransferase.

	**Total events**		**HCC**	
	**Univariate analysis (p-value)**	**Multivariate analysis (p-value)**	**Univariate analysis (p-value)**	**Multivariate analysis (p-value)**
**Obesity**	0.8688	-	0.7752	-
**Diabetes**	0.3276	-	0.4023	-
**Hypertension**	0.7504	-	0.5828	-
**CHD**	0.6360	-	0.7040	-
**Age**	0.0345	0.067243	0.0273	0.027323
**GGT**	0.1120	-	0.0865	-
**Low platelet count**	0.0363	0.040265	0.0702	-
**Albumin**	0.3041	-	0.3684	-
**MELD score**	0.7640	-	0.6135	-
**HBeAg status**	0.5210	-	0.5210	-

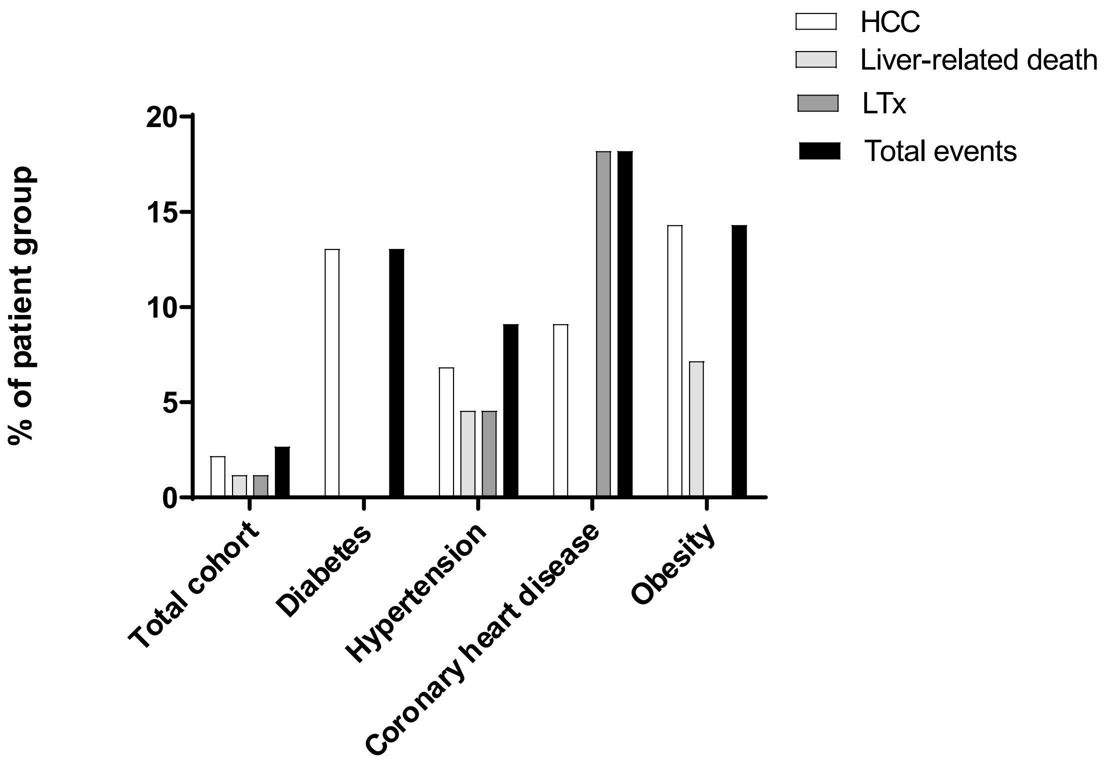

Supplementary Figure S1. Patient outcomes, including hepatocellular carcinoma (HCC), liver-related death, liver transplantation (LTx) and total events, are indicated for the metabolic subgroups: diabetes, hypertension, coronary heart disease (CHD) and obesity. No events occurred in the NAFLD group.
